# Effect of sp^3^ Content on Adhesion and Tribological Properties of Non-Hydrogenated DLC Films

**DOI:** 10.3390/ma13081911

**Published:** 2020-04-18

**Authors:** Chao Li, Lei Huang, Juntang Yuan

**Affiliations:** Jiangsu Engineering Laboratory of High-End Equipment Manufacturing Technology, Collaborative Innovation Center of High-End Equipment Manufacturing Technology, Nanjing University of Science and Technology, str. Xiaolingwei 200, Nanjing 210094, China; michaellee32@163.com (C.L.); mc106@mail.njust.edu.cn (J.Y.)

**Keywords:** non-hydrogenated amorphous carbon, sp^3^ bonding carbon, Ti6Al4V titanium alloy, wear mechanism

## Abstract

Non-hydrogenated diamond-like carbon (DLC) films with various ratios of sp^3^/sp^2^ were prepared on cemented carbide YG8 with DC magnetron sputtering technology. A pure graphite target was selected as the carbon source. Before DLC deposition, a surface etching pretreatment was carried out by mid-frequency magnetron sputtering method, using Ti atoms to improve adhesion strength. The ratios of sp^3^/sp^2^ were adjusted by bias voltages. In order to investigate the effect of the ratio of sp^3^/sp^2^ on adhesion and tribological properties, Raman spectra, XPS spectra, adhesion scratch test and ball-on-disk dry friction tests were applied. The results indicated that the ratio of sp^3^/sp^2^ fluctuated with bias voltage, increasing in the range of 0.74 to 0.98. The adhesion strength decreased from 31.5 to 18.4 N with the increasing ratio of sp^3^/sp^2^, while the friction coefficient rose in DLC-Si3N4 and dropped in DLC-Ti6Al4V. For DLC-Ti6Al4V, the oxidation of Ti6Al4V had a greater influence than graphitization of DLC. The hard oxides of Ti6Al4V broke the graphite transfer layer leading to a high friction coefficient. The wear rate was approximately linearly related to bias voltage. The coefficients of the linear regression equation were influenced by different friction materials. The adhesion strength and the friction coefficient were fitted as a function of the ratio of sp^3^/sp^2^.

## 1. Introduction 

Diamond-like carbon (DLC) film is a type of metastable amorphous carbon film, composed of sp^3^ and sp^2^ hybrid carbon atoms. Therefore, DLC films possess high hardness from the diamond and low friction from the graphite [1,2]. The ratio of sp^3^ and sp^2^ bonds is a crucial intrinsic parameter, determining the structure and properties of DLC films [3,4,5]. In the field of mechanics, DLC films are mainly applied to cutting tools and friction components, which require good adhesion and tribological properties [6,7,8]. High normal and shear stresses are the major causes of DLC failures, such as brittle spalling, abrasive wear and adhesive wear [9,10,11,12]. The adhesion strength, friction coefficient and wear rate are the most direct indexes used to reflect the properties of DLC films. 

The ratio of sp^3^/sp^2^ is a determining characteristic in DLC film properties. More sp^3^ bonds can reduce the friction of ta-C films [13]. The hardness and adhesion of hydrogenated DLC film are closely correlated to the ratio of sp^3^ bonding [14,15]. Under oil-lubricated conditions, the hydrogen-free DLC films with lower sp^3^ content show higher friction coefficients. This is because the dangling bonds of DLC coatings which adsorb the oiliness agent to reduce the friction are in proportion to the sp^3^ content [16,17]. For different types of DLC films, the properties change according to sp^3^ content. 

When rubbing against different materials, DLC films show different tribological performances. Some researchers think that this is due to the different hardness of materials, which influences the transfer layer on the friction interface. The transfer layer is formed by the softer material and transfers to the harder material [18,19,20]. However, oxidation of some materials like aluminum alloy has been proven to affect the friction coefficient and wear rate of DLC film [17,21,22,23,24]. 

In previous studies, deposition parameters, including target power, bias voltage, pressure and gas flow, were adjusted to investigate the level of influence on DLC properties in order to achieve the optimization [25,26,27,28]. The adjustment of the bias voltage is a simple and stable method to obtain DLC with various properties [29,30,31,32]. High substrate bias voltage produces better adhesion to DLC films but also leads to lower hardness and poor wear resistance [33]. DLC layers prepared under bias voltage of 80 and 160 V have a hardness difference of about 5 GPa, so multilayer DLC films can be made up by alternating a hard and a soft DLC monolayer [34]. Although the research substances are similar, the influence of bias voltage differs because of the difference in composition and structure of DLC films, such as the ratio of sp^3^/sp^2^. 

In this paper, the non-hydrogenated DLC films were prepared on cemented carbide YG8 using DC magnetron sputtering technology. With the ratio of sp^3^/sp^2^ in a certain range, the effects of the ratio of sp^3^/sp^2^ on the adhesion and tribological properties of DLC films were studied quantitatively. 

## 2. Experimental Details

**Figure 1:**
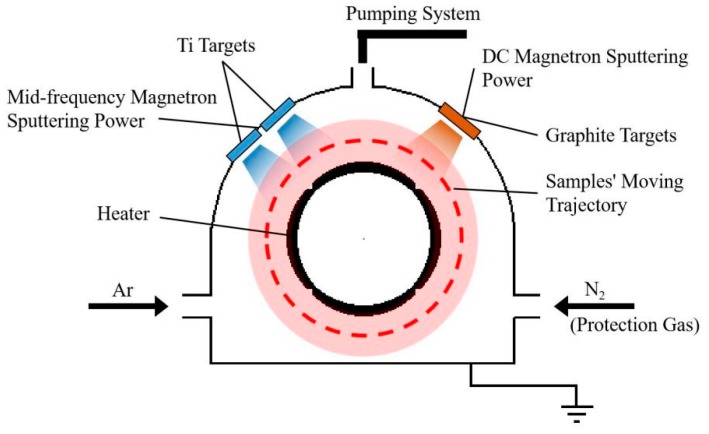
Schematic diagram of MC-hybrid coating system.

Non-hydrogenated DLC films were prepared in an MC-hybrid coating system equipped with two 99.99% purity titanium targets and a 99.99% purity graphite target. Schematic diagram of the coating system was shown in Figure 1. Polished cemented carbide YG8 samples were chosen as substrates and underwent thorough ultrasonic cleaning in acetone, deionized water and ethanol for 15 min. The size of the depositing surface was 10 mm × 10 mm. Before deposition, the substrates were heated to 80 °C. The samples were etched first in an argon flow for 15 min with the bias voltage of −1200 V. The surface pretreatment was carried out by a mid-frequency magnetron sputtering method, using Ti atoms with the current of 4.0 A and the bias voltage of −1000 V for 30 min. The high-energy Ti atoms etched the sample surfaces to the depth of 400 nm. The pretreatments for all the samples were the same. In this study, the DC magnetron sputtering method was used to prepare non-hydrogenated DLC films in an argon atmosphere. During the deposition process, DC voltage was kept at 520 V (DC power was about 600 W). The argon flow was 120 sccm, and the gas pressure in the vacuum chamber was kept at 1.4 Pa. The temperature was kept at 80 °C throughout the deposition process. Different ratios of sp^3^/sp^2^ in the DLC films were obtained by changing the bias voltage from −175 to −300 V. In order to improve the adhesion strength, every DLC film was composed of 10 DLC layers, and each layer was deposited for 15 min. After the deposition process, nitrogen was introduced into the chamber as a protection gas in the cooling stage to avoid oxidation of the samples. Some of the parameters of the DLC films are shown in Table 1. The thickness was the thickness of DLC layers and did not include the depth of surface pretreatment. The surface roughness R_a_ was measured five times on each sample, and each sample length was 200 μm. The average roughness of each sample is shown in Table 1.

The surface and worn morphology were characterized by scanning electron microscope (SEM, FEI Quanta, Hillsboro, OR, USA). The composition of DLC films was characterized by micro-Raman spectrometer (Renishaw in Via, London, UK). The ratio of sp^3^/sp^2^ was measured by X-ray photoelectron spectroscopy (XPS, PHI QUANTERA II, Chigasaki, Japan). The film thickness, surface roughness and cross-section profiles of the wear tracks were measured by stylus profiler (Dektak XT, Bruker, Billerica, MA, USA). The adhesion strength was measured by a coating-adhesion scratch tester (WS-2005, manufacture, city, country), which provided a linear load from 0 to 40 N with a diamond indenter. The scratch morphology was imaged by metallographic microscope. The chemical composition in the wear tracks was examined by energy-dispersive spectroscopy (EDS, Qxford, Oxfordshire, UK). The tribological properties were tested by a reciprocating ball-on-disk tester (UMT-2, Bruker), which reports the friction coefficient automatically, under dry friction conditions with the ambient environment at 25 °C and 30% RH. The friction tests were performed with a constant load of 3 N, velocity of 8 mm/s and time of 20 min, against the Si3N4 and Ti6Al4V balls. The wear rates of DLC films were calculated from the cross-section profiles of the wear tracks. 

## 3. Results and Discussion

### 3.1. Raman and XPS Spectra

**Figure 2:**
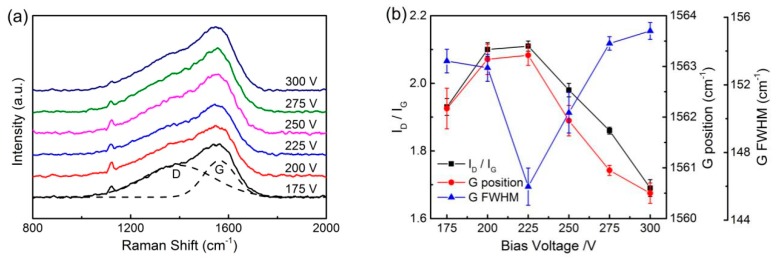
(**a**) Raman spectra of DLC films with various ratios of sp^3^/sp^2^. (**b**) Gaussian fitting results for Raman spectra.

Figure 2 shows the Raman spectra results of the non-hydrogenated DLC films with different ratios of sp^3^/sp^2^ bonds. Figure 2a shows the typical features of amorphous carbon film: namely, a wide asymmetric peak called a G peak appeared at around 1560 cm^−1^, and an inconspicuous shoulder peak called a D peak appeared at around 1390 cm^−1^. Commonly, the content of sp^3^ bonding carbon increases with the ratio of the intensities of the D and G peaks (I_D_/I_G_) decreasing, G peak position moving towards a lower wave number and the full width at half maximum (FWHM) of G peak increasing [4]. Figure 2b shows the variation curves of the I_D_/I_G_, G peak position and G peak FWHM. With the negative bias voltage increasing from 175 to 300 V, the I_D_/I_G_ rose from 1.93 to 2.11 and then dropped to 1.69. At the same time, the G position shifted from 1562.17 to 1563.22 cm^−1^ and then to 1560.5 cm^−1^, while the G peak FWHM decreased from 153.41 to 145.98 cm^−1^ and then increased to 155.19 cm^−1^. This shows that the sp^3^ content considered here decreased when the negative bias voltage rose to 225 V and then increased when the negative bias voltage rose to 300 V. 

**Figure 3:**
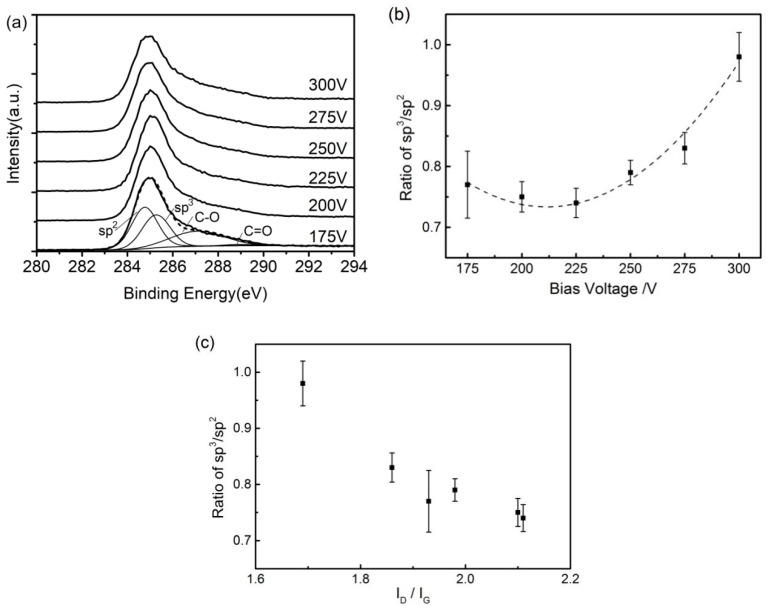
(**a**) X-ray photoelectron spectroscopy (XPS) spectra of DLC films with various ratios of sp^3^/sp^2^. (**b**) Ratio of sp^3^/sp^2^ as a function of bias voltages. (**c**) Ratio of sp^3^/sp^2^ with I_D_/I_G._

The ratios of sp^3^/sp^2^ were measured by X-ray photoelectron spectroscopy. The XPS profiles together with the peak positions of sp^3^, sp^2^, C−O and C=O bonds are shown in Figure 3a. A fitting operation was performed to express the peak value with the fitting rate, showing that the FWHM of sp^3^ and sp^2^ peaks were 1.45 and 1.35 eV, respectively, and the position difference between sp^3^ and sp^2^ peaks was 0.5 eV [35,36]. The ratios of sp^3^/sp^2^ ranged from 0.74 to 0.98. Figure 3b shows the polynomial fitting result of the ratio of sp^3^/sp^2^ on bias voltage. The relationship was expressed by the following equation:

(1) $$R=2.0904-0.0128v+3.0346\times 10^{-5}v^{2}$$

where *R* represents ratio of sp^3^/sp^2^ and *v* stands for bias voltage. The coefficients were determined by the other deposition parameters and the equipment conditions. The coefficient of determination (R^2^) was 0.97, and this equation fitted well against the changing ratios of sp^3^/sp^2^. The changes of sp^3^ content by XPS and by Raman spectra were consistent, as the ratio of sp^3^/sp^2^ shows a downward trend with I_D_/I_G_ in Figure 3c. 

According to previous studies, negative bias voltage has always been a major parameter in assisting deposition of DLC films. In DC magnetron sputtering deposition, negative bias voltage promoted the processes of both the sputtering (to targets) and the etching (to substrates). However, it had effects on two aspects: on the one hand, the increase in bias voltage improved sputtering efficiency by assisting argon ionization to produce more argon ions, which accelerated the argon ions to bombard the graphite target. Due to the higher energy of argon ions, the carbon particles sputtered out of the graphite target contained more and larger graphitic clusters, which decreased the sp^3^ content. On the other, the increase in bias voltage also intensified the collision of the high-energy argon ions with graphitic clusters. This made the graphitic clusters smaller and broke the π bonds, which resulted in an increase of the sp^3^ content. In this paper, sputtering particles by DC magnetron sputtering deposition had low energy, because the energy of sputtering ions mainly offsets high ionization energy (11.26 eV) of the carbon atoms in the graphite target [37,38]. When bias voltage increased to 225 V, argon ions had low energy and broke few graphitic clusters. The increase in bias voltage assisted the sputtering process more significantly and the sp^3^ content decreased. When the bias voltage increased to 300 V, the argon ion had higher energy to extensively break graphitic clusters. Smaller graphitic clusters, which led to more disorder in the graphitic phase in DLC films, and the breakage of more π bonds, contributed to the increase of the content of sp^3^ bonding carbon. 

### 3.2. Adhesion Strength

**Figure 4:**
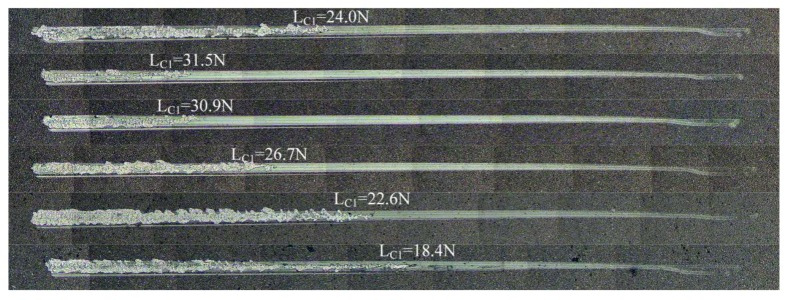
The scratch morphology of DLC films.

The adhesion strength of DLC films was measured by scratch test. The scratch tester provided linear load from 0 to 40 N with a diamond indenter. The critical load (L_C1_) was chosen as the adhesion strength, corresponding to the point at which the fracture first appeared on DLC films [25]. Each sample was measured three times, and the average values, excluding outliers, were recorded as the critical loads. Figure 4 shows scratch morphology of the DLC films. The film failure was classically caused by brittle spalling: when the load was beyond L_C1_, massive spalling appeared quickly, and there were no cracks at the scratch edge. Because of the low power density of the DC power supply, many π bonds could not be broken, and sputtering particles contained quantities of graphite clusters. Graphite microcrystal formations on the DLC films led to the film structure changing towards graphite rather than diamond. This reduced the structural strength against vertical load.

**Figure 5:**
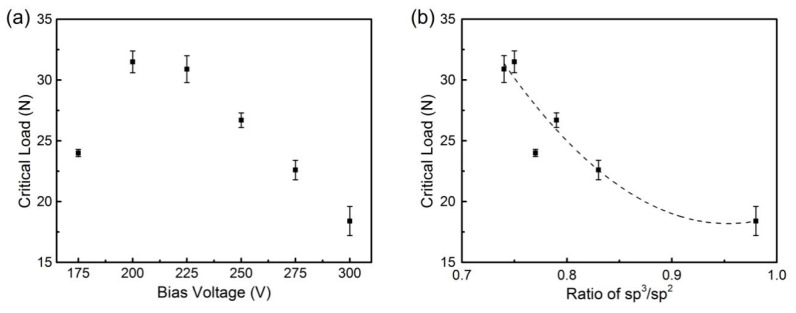
(**a**) Critical loads of DLC films under different bias voltages. (**b**) Critical loads as a function of the ratio of sp^3^/sp^2.^

The adhesion strength increased at first and then decreased with the increase of bias voltage, as shown in Figure 5a. Sample 2 showed the highest adhesion strength (31.5 N), and the adhesion strength decreased to 18.4 N in Sample 6. The rate of change of the adhesion strength was similar to that of I_D_/I_G_. Figure 5b shows the critical loads corresponding to ratios of sp^3^/sp^2^ and the fit of the results. The following equation fitted the relationship of L_C1_ and ratios of sp^3^/sp^2^:

(2) $$L_{C1}=281.6467-552.8349r+290.0209r^{2}$$

where *L_C1_* and *r* represent the critical load and ratio of sp^3^/sp^2^, respectively. R^2^ of the fitting curve was 0.91. Coefficients were mainly related to the substrate and deposition conditions, including material characteristics, transition layers, pretreatment and deposition parameters. The pretreatment used in this paper exposed the surface WC crystals and brought higher chemical activity, which helped to improve the adhesion strength.

Non-hydrogenated DLC films had poor toughness, and failures due to brittleness were frequent. Higher diamond-phase carbon content further reduced the toughness of DLC films, so the adhesion decreased with the increase of sp^3^. In addition, higher bias voltage led to higher energy of argon ions etching the depositing surface. It decreased the thickness of the DLC films, but made the DLC films more compact. This increased compactness did not improve the adhesion strength [34]. 

### 3.3. Tribological Properties

**Figure 6:**
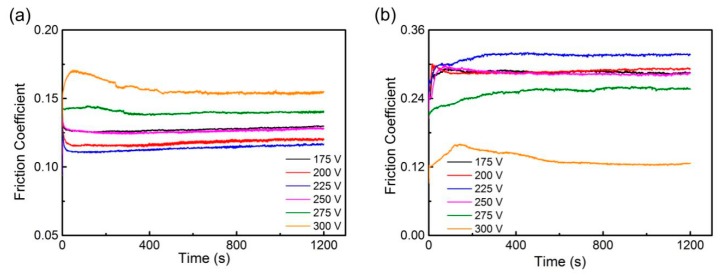
Friction coefficient waveforms against: (**a**) Si3N4; (**b**) Ti6Al4V.

The tribological properties were tested using the reciprocating ball-on-disk test under dry friction conditions on a friction and wear tester (UMT-2, Bruker), against Si3N4 and Ti6Al4V balls. Friction coefficients were measured automatically by the tester device. Figure 6 shows the waveforms of the friction coefficients of all the samples during the tests. The order of the friction coefficients against Si3N4 balls was exactly opposite to the order against Ti6Al4V balls. Sample 3 under bias voltage of 225 V showed the lowest friction coefficient (0.116) against Si3N4 and Sample 6 under bias voltage of 300 V showed the highest (0.155). The friction coefficients against Ti6Al4V were generally higher. Only Sample 6 performed similarly, but it had an even lower friction coefficient of 0.126. The friction coefficient of Sample 3 was 0.316. 

**Figure 7:**
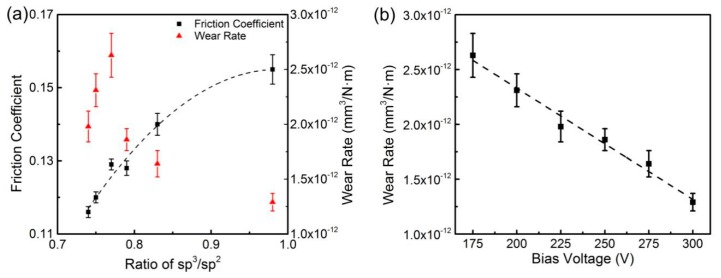
(**a**) Friction coefficients against Si3N4 as a function of ratio of sp^3^/sp^2^ and wear rate; (**b**) wear rate against Si3N4 as a function of bias voltage.

**Figure 8:**
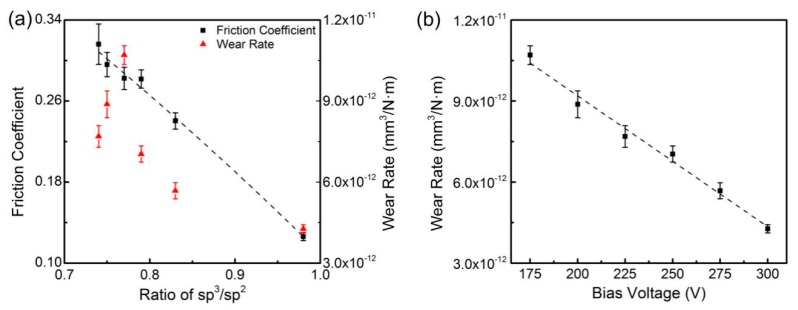
(**a**) Friction coefficients against Ti6Al4V as a function of the ratio of sp^3^/sp^2^ and wear rate; (**b**) wear rate against Ti6Al4V as a function of bias voltage.

The friction coefficients and wear rates of DLC films corresponding to ratios of sp^3^/sp^2^ are shown in Figure 7a and Figure 8a. The friction coefficient against Si3N4 increased with ratios of sp^3^/sp^2^ increasing, but the friction coefficient against Ti6Al4V decreased. The friction coefficients were fitted with the following equations:

(3) $$f_{Si3N4}=-0.4498+1.2248r-0.6200r^{2}$$

(4) $$f_{Ti6Al4V}=0.6879-0.3258r-0.2523r^{2}$$

where *f_Si3N4_* and *f_Ti6Al4V_* represent friction coefficients against Si3N4 and Ti6Al4V, respectively, and *r* represents the ratio of sp^3^/sp^2^. Coefficients were determined by the substrate conditions and friction test conditions, such as temperature, humidity and test parameters. In addition, the parameters of Function (4) were influenced by the oxidation in friction process. R^2^ values of the fitting curves against Si3N4 and Ti6Al4V were 0.967 and 0.995, respectively. Neither wear rate had definite correlation with the ratios of sp^3^/sp^2^ from Figure 7a and Figure 8a, but the wear rates both decreased approximately linearly as bias voltage increased. The wear rates and bias voltages were fitted by linear regression as shown in Figure 7b and Figure 8b with the following equations:

(5) $$w_{Si3N4}=4.3575\times 10^{-12}-1.014\times 10^{-14}v$$

(6) $$w_{\mathrm{Ti}6\mathrm{Al}4V}=1.8889\times 10^{-11}-4.8457\times 10^{-14}v$$

where *w_Si3N4_* and *w_Ti6Al4V_* represent wear rates against Si3N4 and Ti6Al4V, respectively, and *v* represents bias voltage. Intercepts and coefficients were determined by the substrate conditions and friction test conditions, such as temperature, humidity and test parameters. R^2^ values of the fitting curves against Si3N4 and Ti6Al4V were 0.98 and 0.98, respectively. As reported, higher bias voltage contributes to achieve better compactness, which can enhance the wear resistance of DLC films [31]. The relationship between wear rates and bias voltages confirmed that high bias voltage brought good wear resistance to DLC films. 

**Figure 9:**
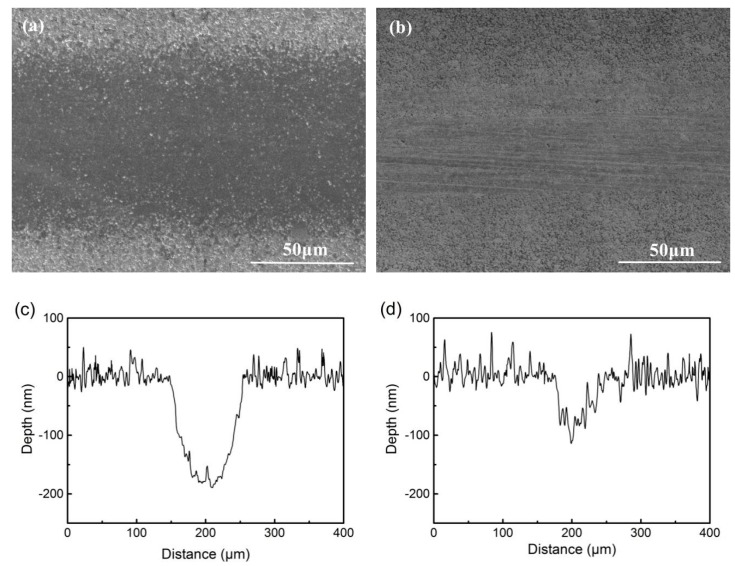
The wear scratch morphology and cross-section of DLC-Si3N4: (**a**,**c**) Sample 3; (**b**,**d**) Sample 6.

Si3N4 is a type of ceramic material with high hardness and good chemical stability. During the process of rubbing against Si3N4 balls, the graphitization of the DLC films occurred on the friction interfaces. The graphitic transfer layers, which are beneficial to decreasing the friction coefficients, were formed and transferred onto the surfaces of Si3N4 balls. For the DLC films in this paper, graphitization was a process of sp^3^ bonding carbon turning to sp^2^ bonding carbon and then to graphite microcrystal. The graphitization of the DLC films with more sp^2^ bonding carbon was faster because of the higher content of graphitic clusters. Sample 6 had the lowest content of sp^2^ bonding carbon and showed the highest friction coefficient. Figure 9 shows the morphology of the wear scratches and cross-sections at middle positions of Sample 3 and Sample 6. Abrasive wear was found in the wear scratch of Sample 6, and the wear scratch of Sample 3 was smoother in Figure 9. This indicated that the carbon separated from Sample 6 did not act as a lubricant on the friction surface.

DLC films have different tribological performances against various materials which have different hardness and chemical stability [21,22]. Compared to Si3N4, Ti6Al4V has lower hardness and is more easily oxidized. The change rate of the friction coefficient of DLC-Si3N4 was almost reverse that of DLC-Ti6Al4V. Graphite-phase-carbon increasing is beneficial to decrease the friction coefficient, and Si3N4 is difficult to oxidize. Therefore, the friction coefficient against Si3N4 changes with the content of sp^3^ bonding carbon, without the interference of oxidation. The oxidation of Ti6Al4V affected the friction coefficient more obviously than the change in the sp^3^ bonding carbon. When the outermost Ti6Al4V layers were removed, inner atoms of Ti and Al with good chemical activity were exposed to the atmosphere. These atoms were quickly oxidized into Al_2_O_3_ and other oxides. These hard oxides aggravated the wear on the DLC film and resulted in the generally higher friction coefficient. The DLC films prepared at lower bias voltage had higher wear rates, and Ti6Al4V was easier to be abraded than Si3N4. This suggests the contact areas between the DLC and Ti6Al4V on the friction interface were larger. The oxidation on these samples was more severe, and it led to the reverse in order of the friction coefficients found against Si3N4.

The average element content in wear debris and on friction interfaces of DLC films and Ti6Al4V balls are listed in Table 2. There were few Ti or Al atoms left in the wear scratches on DLC films, while carbon accounted for about 22.93% of the elements in wear marks on Ti6Al4V balls. Carbon comprised about 75.46% of the wear debris, which suggested that abrasion of DLC films was far greater than abrasion of Ti6Al4V balls in the process of friction. The carbon left on Ti6Al4V balls provided evidence of the existence of the graphitic transfer layers. Oxygen was present in about 45.65% of wear marks and 16.90% of wear debris. This indicated that oxidation occurred on the friction interface of Ti6Al4V balls. The results of oxidation contained hard oxides (e.g., Al_2_O_3_), which broke the graphitic transfer layers and resulted in a high friction coefficient. 

**Figure 10:**
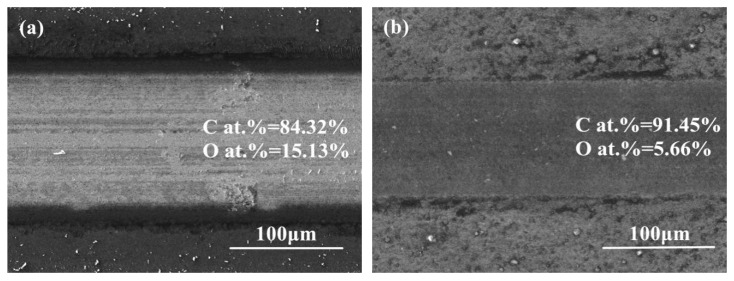

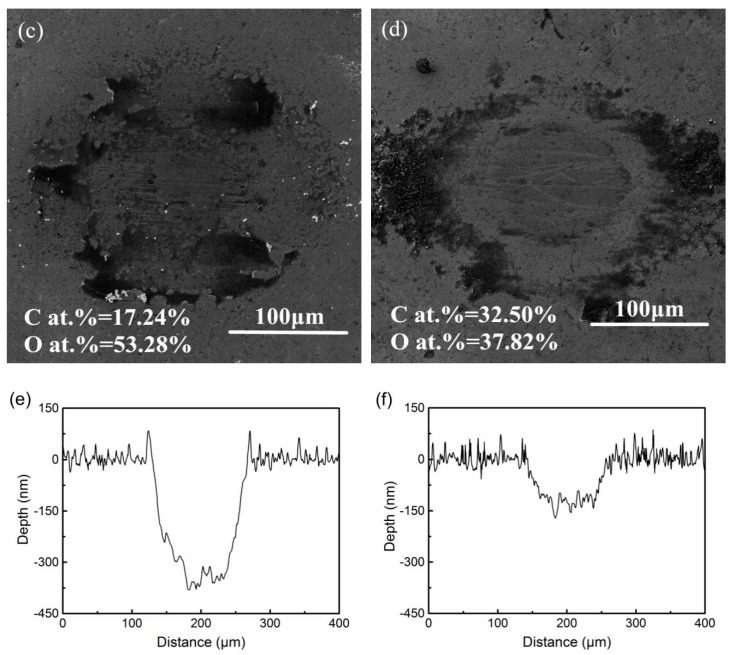
The wear scratch morphology and cross-section of DLC-Ti6Al4V: (**a**,**c**,**e**) Sample 3; (**b**,**d**,**f**) Sample 6.

Sample 3 had the highest friction coefficient (0.316). There were deep furrows in the wear scratches, with some wear debris accumulating in the furrows in Figure 10a. The wear scratch of Sample 6 was smooth and narrow in Figure 10b,f. The friction coefficient of Sample 6 was only 0.126. The carbon and oxygen contents in the DLC wear scratches and in Ti6Al4V wear marks of Sample 3 and Sample 6 are shown in Figure 10. A higher presence of oxygen on the Ti6Al4V ball of Sample 3 represented greater oxidation of Ti6Al4V. Comparing the wear mark on Ti6Al4V ball of Sample 3 with that of Sample 6 in Figure 10c,d, there were many scratches in the wear mark of Sample 3, and the graphitic transfer layer was blurred, while the graphitic transfer layer of Sample 6 was almost complete. The blurred graphitic transfer layer showed that the transfer layer existed at the early stage but was broken later by friction. This confirmed that the product of Ti6Al4V oxidation did not become part of the transfer layer but existed as oxides in the wear debris. The hard oxide debris was responsible for the high friction coefficient and poor wear resistance of Sample 3. The oxygen content on Ti6Al4V balls was much higher than that on the DLC films. This was probably because most of the oxide debris was embedded in the transfer layer on the Ti6Al4V surface and the Ti6Al4V was further oxidized after the friction test.

The wear rate of the DLC film was related to bias voltage more significantly than to the ratio of sp^3^/sp^2^. When the bias voltage rose, the argon ions had increased energy to etch the depositing surface and to remove the particles that were combining poorly to the substrate. At the same time, the deposited layer was compressed and obtained better compactness. In addition, the increase of sp^3^ content added diamond phases to DLC films and enhanced the structural strength. The rate of change in wear indicated that the effect of compactness was greater than that of film composition, within a certain range.

## 4. Conclusions

The non-hydrogenated diamond-like carbon (DLC) films with various contents of sp^3^ bonding carbon were prepared on cemented carbide YG8 with DC magnetron sputtering technology. The adhesion strength was improved by pretreatment with Ti atoms, which were used to etch the substrate surface. The ratio of sp^3^/sp^2^, measured by XPS spectra, was in the range of 0.74 to 0.98. In this range, the influence of ratio of sp^3^/sp^2^ on adhesion and tribological properties of DLC films was investigated. The ratio of sp^3^/sp^2^ was adjusted by bias voltage and the relationship was fitted as a function. Higher bias voltage increased the sputtering efficiency to the graphite target and enhanced the etching effect to the depositing surface, which contributed to the increase of sp^3^ content. The adhesion strength decreased because higher sp^3^ content reduced the toughness of the DLC film. A function was presented to describe the relationship between adhesion strength and the ratio of sp^3^/sp^2^. In DLC-Si3N4 friction, more sp^2^ bonding carbon promoted the formation of a graphite transfer layer in the friction interface to decrease the friction coefficient. In DLC-Ti6Al4V friction, the oxidation of Ti6Al4V created hard oxides which broke the graphite transfer layer and led to definite abrasive wear. The wear rate of DLC film showed a linearly decreasing correlation with bias voltage, rather than with the ratio of sp^3^/sp^2^.

## Figures and Tables

**Table 1:** Parameters of different diamond-like carbon (DLC) films.

Sample	Bias Voltage (V)	DLC Thickness (nm)	Surface Roughness (nm)	Ratio of sp^3^/sp^2^ Content
1	175	645	14.82	0.77
2	200	611	18.63	0.75
3	225	559	15.97	0.74
4	250	472	17.28	0.79
5	275	378	18.17	0.83
6	300	337	20.70	0.98

**Table 2:** Element contents on the friction interfaces.

	Wear Scratch on DLC (%)	Wear Mark on Balls (%)	Wear Debris (%)
C	87.86	22.93	75.46
O	10.35	45.65	16.90
Al	0.29	4.32	1.92
Ti	1.50	27.10	5.72
